# Early stage chronic kidney disease: a paradigm for diffuse fibrosis and clinical progression

**DOI:** 10.1186/1532-429X-16-S1-O66

**Published:** 2014-01-16

**Authors:** Nicola C Edwards, William E Moody, Mengshi Yuan, Shonagh Flanagan, Charles J Ferro, John N Townend, Richard P Steeds

**Affiliations:** 1 Cardiovascular Medicine, University of Birmingham, Birmingham, UK; 2 Cardiology, Queen Elizabeth Hospital, Birmingham, UK

## Background

Early stage chronic kidney disease (CKD) is an under recognised, highly prevalent cardiovascular (CV) risk factor. Despite a clustering of conventional atherosclerotic risk factors, there is a disproportionate rate of sudden cardiac death, heart failure and stroke rather than myocardial infarction. It is hypothesised that non-atherosclerotic processes, including left ventricular (LV) hypertrophy and fibrosis account for most of the excess CV risk.

## Methods

In total, 30 patients (mean age 57 +/- 8 years) with CKD stage 2-4 (mean GFR 58 ± 22 ml/min/1.73 m2) with no history of cardiovascular disease (mean BP 129 mmHg ± 13/70 mmHg ± 11) or diabetes were compared with age and gender matched controls. All subjects underwent cardiac MRI (1.5T Siemens Avanto) for assessment of LV volumes, myocardial deformation (SPAMM tagging) and aortic distensibility. Late gadolinium enhancement (LE) and T1-mapping using a modified look-locker inversion recovery sequence (MOLLI) before and 15 minutes post gadolinium (0.1 mmol/Kg) for myocardial extracellular volume (ECV) were performed. Global ECV was the average measure from basal and mid short axis slices excluding LGE indicative of infarcted myocardium.

## Results

Global ECV was increased in CKD (0.30 ± 0.05 vs. 0.27 ± 0.03, p < 0.05) with associated reductions in long axis systolic deformation (strain 14.8% ± 2 vs. 17.1% ± 2, p < 0.05), early relaxation (SR e' 0.50 ± 0.2 vs. 0.67 ± 0.2, p < 0.05) and MAPSE (13 mm ± 2 vs. 17 mm ± 3, p < 0.05). Aortic distensibility was reduced in CKD (Table 1). There were no differences in LV end-diastolic volumes, end-systolic volumes, LV mass index or LV EF (Table 1). Three patients had LGE at the RV insertion points and four patients had diffuse mid-wall LGE in a non-coronary artery territory distribution. NT-proBNP was not increased (median 89 ng/L, IQR 150). ECV did not correlate with conventional CV risk factors including systolic blood pressure, cholesterol or urinary albumin:creatinine ratio.

**Table 1:** CMR parameters of structure and function

	CKD (n = 30)	Controls(n = 30)
LVEDVi (ml/m2)	70 (14)	64 (13)
LVESVi (ml/m2)	21 (7)	19 (7)
LV mass index (g/m2)	63 (14)	58 (13)
LVEF (%)	71 (6)	73 (6)
MAPSE (mm)	13 (2)	17 (3)*
LA volume index (ml/m2)	45 (12)	33 (10)*
Peak longitudinal systolic strain (%)	14.8 (2.0)	17.1 (2.0)*
Peak longitudinal systolic strain rate (s-1)	0.71 (0.35)	0.91 (0.23)
Early longitudinal diastolic strain rate (s-1)	0.50 (0.17)	0.67 (0.24)*
Aortic distensibility (mmHg-1)	2.20 (1.56)	3.7 (1.67)*

Data = mean (SD), *p < 0.05

## Conclusions

Diffuse LV fibrosis is increased in early CKD with associated reductions in aortic distensibility and abnormal regional systolic and diastolic function. An increase in ECV detected by T1 mapping might be a key intermediate phenotype and follow-up studies are required to determine if it is predictive of impaired LV systolic function, heart failure events and mortality in CKD.

## Funding

Nil.

